# An Assessment of Prisoner Reentry, Legal Financial Obligations and Family Financial Support: A Focus on Fathers

**DOI:** 10.3390/ijerph18189625

**Published:** 2021-09-13

**Authors:** Andrea N. Montes, Danielle Wallace, Chantal Fahmy, Abigail Henson, Alyssa W. Chamberlain, Leah A. Jacobs

**Affiliations:** 1 School of Criminology and Criminal Justice, Watts College of Public Service and Community Solutions, Arizona State University, 411 N. Central Ave., Phoenix, AZ 85004, USA; Danielle.wallace@asu.edu (D.W.); Abigail.henson@asu.edu (A.H.); Alyssa.chamberlain@asu.edu (A.W.C.); 2 Department of Criminology & Criminal Justice, College for Health, Community and Policy, University of Texas at San Antonio, 501 West Cesar E. Chavez Blvd., San Antonio, TX 78207, USA; Chantal.fahmy@utsa.edu; 3 School of Social Work, University of Pittsburgh, 4200 Fifth Ave, Pittsburgh, PA 15260, USA; leahjacobs@pitt.edu

**Keywords:** financial sanctions, legal financial obligations, familial support, prisoner reentry, collateral consequences

## Abstract

Scholars have found that family support is an important facilitator of successful reentry from prison to the community. At the same time, they have argued that owing court-ordered fines or fees, also called legal financial obligations (LFOs), can act as an additional barrier to reentry, especially for parents. There remains a need to test how LFOs impact the financial support formerly incarcerated parents receive from their families. The current study responds to this gap by employing logistic regression analyses of the Serious and Violent Offender Reentry Initiative (SVORI) data to test whether owing court fees is associated with formerly incarcerated fathers’ (1) perceptions of available financial support from family and (2) receipt of financial support from family. We find that owing court fees is not associated with perceptions of available financial support. However, owing court fees has a positive, statistically significant association with receiving financial support from family during the first three months after prison release. This relationship remains after accounting for whether the person owes child support or sees their children monthly. Our results suggest that LFOs may create a greater need for financial support among formerly incarcerated fathers, making the financial challenges of reentry a consequence not just for those who were incarcerated but for their loved ones as well.

## 1. Introduction

Reentry to the community from prison has become a common experience in the United States. In 2019, an average of 1665 individuals were released from a state or federal prison every day, with more than 70% of these individuals under some form of conditional release [1]. These conditions can include, among others, mandatory drug tests, frequent check-ins with a parole officer, and participation in community programs [2]. An additional condition can entail paying court-ordered fines or fees, also called legal financial obligations (LFOs). Though estimates of a typical amount owed vary, LFOs can be substantial. One study found that among the formerly incarcerated individuals surveyed, they each owed an average of $13,607 to the courts when leaving prison [3]. Another study of returning citizens across three states found that over half the sample owed LFOs [4]. The amounts owed ranged from $10 to $13,200, with a median amount of $260. A third study, which analyzed the State Court Processing Statistics, found that among individuals sanctioned with fines, the median amount imposed was $506; and among individuals sanctioned with restitution, the median amount imposed was $400 [5].

Criminal justice debts can compound already-existing barriers to successful reentry. When individuals prioritize paying their LFOs, it means they have less money to put toward costs associated with successful reentry, such as securing stable housing and reliable transportation [2,6,7]. These needs are important for all returning citizens but can carry extra urgency for parents who will have children living with them or who are seeking to provide support for their children’s needs. For example, LFOs may compromise a parent’s ability to pay child support [8].

Scholars have hypothesized that LFOs may harm formerly incarcerated individuals’ families who are already burdened with providing critical support during their loved one’s reentry [3,8]. Families who live in poverty and have an incarcerated loved one spend much of their annual income on that person’s debts, fees, and supplies [9]. Upon the individual’s release to the community, families often find themselves in the position of having to provide additional support. One study found that 80% of formerly incarcerated individuals lived with a family member after release from prison [10]. The same study found that over 75% of released individuals had received financial support from their families. If providing this assistance strains relationships between family members during this critical period of reentry, it can cause further harm not just to the individual but to the family as well, especially for parents who are trying to navigate co-parenting responsibilities [11].

It is likely that individuals who have LFOs rely on family members, whether directly or indirectly, to meet their financial obligations. Indeed, scholars have made exactly this argument [12]. Such arguments have stemmed primarily from findings presented in descriptive and qualitative studies of court fees [5]. Researchers have not yet used quantitative methods to investigate the effect owing court-ordered fines and fees has on formerly incarcerated parents and their families. The current study responds to this gap in the literature.

In the current study, we argue that LFOs can exacerbate the financial burden experienced by families of formerly incarcerated fathers. To this end, we use data from the Serious and Violent Offender Reentry Initiative (SVORI) to examine whether having LFOs at prison release influences the perceived and actual financial support families give to their formerly incarcerated loved ones. These data allow for a particular focus on fathers, a focus that is warranted given that the majority of people released from prison are men (see, [1]). In addition, we focus on the first three months after prison release, a period that scholars have found to be especially challenging for individuals reentering society (e.g., [13,14,15]). In what follows, we first introduce what is known about LFOs with a focus on their different forms and who is likely to be assigned this sanction. We also discuss how LFOs can alter the reentry experience, including familial relationships, with a specific focus on this association for parents. Second, we describe this study’s research questions and the data and methods used to investigate them. Third, we present the results from these analyses. Finally, we conclude with a discussion about this study’s implications for scholarship and policy.

### 1.1. Legal Financial Obligations: What Are They and Who Receives Them?

The last several decades are characterized by a pursuit of justice through increasingly punitive sanctions and an unprecedented expansion of the criminal justice system. In 1985, for example, there were approximately 2.9 million individuals under correctional supervision in the United States (U.S.) [16]; in 2018, over 6.4 million U.S. adults were under correctional supervision [17]. There has also been an expansion of other sanctions, including court-ordered fines and fees. Henricks and Harvey estimated that in 2012, state and local revenue from monetary punishments was $15.7 billion, a 650% increase from 1977 [18].

Rather than acting as a replacement for other punitive sanctions, LFOs are often imposed as an additional punishment and can be inflicted at each stage of the criminal justice process [19]. LFOs can include fines, fees, restitution, or a combination of the three. Fines are a court-ordered punishment for breaking the law. An example is being assigned a fine for speeding or riding a bus without a ticket. Restitution is money owed to help pay for the financial cost the crime inflicted on the victim (e.g., cost of stolen property). Fees are costs associated with “use” of the criminal justice system. Examples include the use of a public defender, probation and parole supervision, and room and board at a jail or prison [4]. In some jurisdictions, individuals who cannot immediately pay LFOs may be charged “poverty penalties”, such as payment plan fees, interest on the amount owed, and late fees [20] (p. 1). If they are unable to pay them over the longer term, they can be reincarcerated.

LFOs are a common correctional sanction. Eighty-four percent of individuals on probation receive a financial sanction, often in the form of a supervision fee, and 66% of convicted individuals are subjected to both a carceral sentence and LFOs [6,21]. There is mixed evidence on whether individual-level factors, such as race or crime type, predict LFO assignment [4,22]. However, a recent study found that LFOs are more likely to be issued as a sanction in communities that have more Black residents, a higher percent of noncitizen residents, greater police spending, and have recently undergone tax composition reconfiguration [18].

### 1.2. Legal Financial Obligations and Prisoner Reentry

Scholars have argued that LFOs can compound disadvantage [23]. To illustrate, one study found that approximately 20% of individuals on parole have legal debts that are greater than their monthly income [10]. Another study found that the median amount of LFOs imposed on individuals convicted of felonies in Washington was $7234 [6]. At the time of the study, the average amount owed after accounting for payments made was $5254. When Harris and her colleagues [6] compared these legal debts to Western’s [24] estimates of formerly incarcerated individuals’ annual income they concluded that, “White, Hispanic, and Black men owed 60%, 36%, and 50%, of their annual incomes in legal debt, respectively” [6] (p. 1776). This situation highlights the potential for LFOs to exacerbate the barriers formerly incarcerated individuals, especially Black and Hispanic men, already experience during reentry.

Formerly incarcerated parents may be at an even greater disadvantage economically in part because, in addition to LFOs, child support can accrue while incarcerated. Though child support is different from LFOs, it can compound the difficulties created by LFOs and can act as a barrier to paying off LFOs [25]. A qualitative study conducted with 125 previously incarcerated fathers found that participants had an average child support debt of $36,500 [25]. Another qualitative study conducted with 131 previously incarcerated individuals found that participants’ average legal debt when released from prison increased by over $4700 when child support was added to their court debt [23]. Quantitative studies using data from the Serious and Violent Offender Reentry Initiative, the dataset used in the current study, support these qualitative findings: More than half of formerly incarcerated men with children owed $5000 in child support [26].

The potential collateral consequences of having LFOs are vast. Owing legal debts can lower individuals’ credit scores, which may make it more difficult to pass credit checks [6]. It can also keep someone from having their right to vote restored [20]. When individuals default on their LFOs, it can make them ineligible for federal assistance, such as Temporary Assistance for Needy Families, Supplemental Security Income, and food stamps [20]. It can also lead to arrest, extended periods on community supervision, and reincarceration [20,27]. Legal debts can contribute to anxiety, feelings of hopelessness, anger and pessimism, reinforced dependence, and can perpetuate a criminal identity [23]. Not least, LFOs can keep individuals from being able to obtain basic necessities. One person who owed LFOs told Harris and colleagues: “But sometimes, if I pay my LFO, I don’t have enough [money] left over for food” [6] (p. 1779).

These consequences do not impact all returning citizens equally. As argued by Harris [12] in *A Pound of Flesh*, LFOs disproportionately harm lower-income minority individuals and can further exacerbate the challenges caused by living in poverty. In addition, Black and Hispanic individuals are more likely to be subjected to carceral control [1] and, therefore, the collateral consequences of that punishment. Of particular note is the employment challenges faced by Black men with a criminal record (e.g., [24]). The ubiquity of this discrimination compounded with higher poverty rates can confine Black justice-involved individuals into a continuous cycle of criminal justice involvement and a life of poverty [12,27].

### 1.3. Familial Support during Reentry

One of the factors that can help individuals overcome these barriers to reentry is receiving support from family [10,28,29,30,31,32]. Anywhere between 66% and 92% of people leaving prison depend on their family immediately following release for both emotional (e.g., advice, demonstrations of love) and tangible, or instrumental, support (e.g., assistance with practical tasks) [32]. Familial support extends across multiple domains, including housing, transportation, emotional support, instrumental support, employment, and childcare [30,33,34,35,36,37]. Of particular relevance to the current study, families also provide financial support. Some estimates suggest that more than 92% of formerly incarcerated individuals received cash assistance from family members [29,38]. This support is crucial, especially because many formerly incarcerated individuals have limited resources of their own.

For parents, particularly those of young children, contending with childcare and ongoing custody issues during their incarceration is a notable challenge [28]; one that continues into reentry. Social support from family members who will assist with the caregiving of children during incarceration and post-release is imperative to family reunification and the formerly incarcerated parent’s success generally [28,39]. In addition, some released parents seek to regain custody of their children, which is an additional, time-consuming, and expensive process [37,40]. Once the released person is back in the community, role changes within the family can happen quickly. For instance, the economic well-being of children is generally directly related to their father’s potential employment and earnings upon release [41]. However, formerly incarcerated fathers are less likely to contribute financially to the family. When they do contribute, their contributions average $1300 less per year than similarly situated men who were not incarcerated [41,42]. This situation suggests that, for formerly incarcerated parents especially, support from families may continue to be needed over the long term and well after release.

### 1.4. Legal Financial Obligations and Familial Support

When it comes to LFOs, researchers argue that the adverse impacts of legal debt are experienced by more than just the reentering individual and can create stress and conflict for families who financially assist returning individuals [43]. The majority of families experiencing the incarceration of a parent have limited resources and live in poor communities (see generally, [41,42,44]. When a parent is incarcerated, their family and children simultaneously lose that person’s income and incur costs related to that person’s incarceration.

Families cannot begin their financial or emotional recovery when their released loved one owes court debts. Indeed, one study found that 63% of convicted individuals relied on their family’s help to pay their LFOs [3]. In these cases, family members help to subsidize LFO payments as reentering individuals struggle to attend to their financial debts while also navigating the challenges of reentry [43,45]. Even if they are able to quickly find a job—research suggests most will not [46]—some of that person’s income will have to be put towards debts accrued while incarcerated, including LFOs.

Research shows that debt can cause stress within a family (e.g., [47]), especially for parents [48]. This tension may be especially great for reentering parents whose families may feel that the parent is asking for too much by seeking assistance for themselves and for their children. This situation may be more likely for individuals whose legal debts limit the financial contributions they can make to the needs of their family. Such assertions about how or if LFOs create extra burdens for families rely primarily on descriptive, qualitative, or legal assessments [5]. Quantitative studies could build on this foundational work, empirically testing the relationship between LFOs and family support.

There also remains a need for tests of how legal debts influence the financial support received by formerly incarcerated parents, a group thought to be especially vulnerable to the adverse consequences of LFOs. Of particular importance is the need to account for parental involvement. For example, it may be that a family’s inclination to help a formerly incarcerated loved one who is a parent may be contingent on the efforts made by that person to be involved with their children. To date, there is a considerable gap in knowledge of how parents, and fathers in particular, manage the reentry process and what factors facilitate successful reentry [49].

It is these gaps in the literature to which the current study responds. Specifically, this study answers three research questions with a particular focus on formerly incarcerated fathers:

*RQ1.* Does owing court fees (i.e., LFOs) predict perceptions of available financial support from family?

*RQ2.* Does owing court fees (i.e., LFOs) predict receipt of financial support from family?

*RQ3.* How does the relationship between owing court fees and perceptions and receipt of familial financial support vary when accounting for parental involvement and other family-specific financial costs, such as child support?

The prior literature suggests competing hypotheses. One way to interpret the prior literature is that, compared to those without LFOs, parents with LFOs may be less likely to perceive or receive financial support from family. These individuals and their families will face the competing needs of providing for their children and paying LFOs. Family members may focus their support on the needs of the person’s children rather than the person themselves. However, it may instead be the case that families of formerly incarcerated parents who owe LFOs will provide even greater support to their loved one in an effort to help them meet both their legal obligations and their parental obligations. Because parental status is only one indicator of parental involvement, we anticipate that having parental responsibilities, whether through child support or frequency of contact with children, will account for the effect owing court fees has on familial support. In what follows, we describe the data and methods used to test these hypotheses. We then present this study’s findings.

## 2. Materials and Methods

### 2.1. Data

The current study includes an analysis of two waves of interview data from the adult male sample of the Serious and Violent Offender Reentry Initiative (SVORI). The SVORI was a federally-funded project that sought to enable states to develop programs that would ease released persons’ transition to society and improve their reentry outcomes, such as recidivism and employment [50]. The data collection effort involved conducting detailed interviews with respondents who were incarcerated across 14 facilities in 12 U.S. states. One-and-a-half hour interviews were conducted with individuals approximately one month prior to prison release (baseline), as well as 3, 9, and 15 months post-release [50]. We were particularly interested in the months immediately following release, a time period that scholars have highlighted as critical for successful reentry ([51], see [52]). Consequently, we relied on data collected during the baseline (in-prison) and wave 2 (3 months post-release) interviews. Given that most incarcerated parents are adult men, that the SVORI samples of women and juvenile males are small, and that effect variation likely exists across these subgroups, we focus our analysis on fathers.

### 2.2. Study Variables

All summary statistics discussed in this section are derived from their weighted descriptive statistics.

#### 2.2.1. Dependent Variables

To assess whether LFOs are associated with familial support, we focus on two dependent variables. The first is a dichotomous variable of formerly incarcerated fathers’ perceptions of available financial support from family. Respondents were asked at 3 months post-release (1 = yes; 0 = no), “Is there someone in your family you can turn to for financial support?” As shown in Table 1, nearly 84% of respondents reported that someone in their family could provide financial support should they need it.

The second dependent variable measures whether respondents received financial support from a family member during the first three months of their release. Respondents were asked, “After you were released, how did you support yourself?” with answer choices of “A job”, “Support from your family”, “Support from your friends”, “A government program”, “Illegal income”, and “Some other type of support”. From this variable we created our objective familial financial support outcome (1 = yes, received familial financial support; 0 = no, did not receive familial financial support). Just over 15% of respondents indicated that their families provided them with financial support (Table 1). Notably, this percentage is far lower than the percentage of respondents who perceived having family who *could* provide financial support. Although we do not investigate the reasons for this gap in the current study, it is an area worth future empirical attention.

#### 2.2.2. Independent Variables

To test this study’s primary question about the effect of LFOs on familial support, we used a trichotomous variable collected at wave 1 to measure whether individuals owed court fees upon release. The categories are: does not owe court-ordered fees (=0); owes court-ordered fees (=1); or the respondent was unsure or left blank the court-ordered fees question (=2). A clear limitation of the interview question is that it did not include an “I do not know” option even though it is likely that many individuals in fact did not know whether they owed court fees (Free Our Vote [53], for example, is an organization dedicated to helping individuals learn whether they have LFOs). For this reason, we interpreted missing values, or a lack of response, to indicate either that respondents did not know whether court fees were owed or that they did not answer the question. Each of these categories was turned into a dummy variable when entered into the model; no fees is the reference category. As shown in Table 1, the majority of individuals in the sample owed court fees (54%), approximately one-quarter of respondents did not owe fees (25.8%), and approximately 20% were unsure or did not provide a response.

#### 2.2.3. Control Variables

Related to our third research question, we included control variables that account for parental responsibilities. First, we use a binary variable that indicated whether the respondent owed court-ordered child support (1 = yes; 0 = no); more than one-third of respondents did (36.1%; Table 1). Second, respondents were asked how often they spend time with their minor children. We use a dichotomous measure to indicate whether fathers see their children at least monthly (1 = yes; 0 = no). Over 67% of men in the sample reported seeing their minor children at least monthly (Table 1). Both variables were assessed at wave 2.

Control variables and their descriptive information are detailed in Table 1. We start with sociodemographic variables scholars have highlighted as influencing individuals’ experiences with the criminal justice system and the impacts of those experiences on reentry (e.g., [54,55,56]). We controlled for individuals’ relationship status at wave 2, which was measured by asking respondents if they were currently involved in a steady intimate relationship, including marriage (1 = yes; 0 = no). Approximately 44% answered “yes”. The age of respondents (in years) was accounted for as well. The age variable is zero-centered at age 18, with a mean age of 32.51 years old. Race/ethnicity is captured by a series of dummy variables for Black, Hispanic/Latino/Spanish, other race, and White (reference category). Approximately 47% of the sample identified as Black, 0.8% as Hispanic/Latino/Spanish, 16.6% as other or mixed race, and the remaining 35.5% of respondents identified as White. To account for individuals’ educational attainment, we controlled for whether the respondent had completed high school or received a general equivalency diploma (GED) (1 = yes; 0 = no). In this sample, 62.5% had received a high school diploma or GED. We also control for whether individuals were employed at the wave 2 interview (1 = yes; 0 = no). At that time, 67% reported being employed.

Several carceral and support-related variables were also included as controls. Each of these variables was believed to be related to an individual’s experience with legal debts, as well as their likelihood of receiving familial support (e.g., [5,12,26]). We included a continuous measure of the total number of days incarcerated (logged) during this prison term, as it may be related to a family’s willingness to provide support over the long term. Also included was whether individuals participated in the experimental SVORI reentry programming (1 = yes, 0 = no; 50.9%). Employment support (1 = yes; 0 = no) and friend/family support (1 = yes; 0 = no) were used to indicate how the respondent supported themselves prior to their current incarceration. Approximately 69.2% supported themselves by having a job, while 31.4% were supported by friends and/or family members.

We included two control variables that accounted for aspects of individuals’ health (e.g., [33]). These variables captured other types of needs individuals may have had during the first three months of reentry. These included a dummy variable (1 = yes; 0 = no) that measures if the respondent had any of the following chronic ailments: asthma, diabetes, heart problems, arthritis, back problems, tuberculosis, HIV/AIDS, and hepatitis B or C. Nearly half (48.9%) reported having one of these chronic illnesses or diseases. We also included a dichotomous variable, measured at the 3 month interview, indicating whether they needed drug or alcohol treatment but did not have access to it (1 = yes; 0 = no). Over 20% of respondents in the sample reported having these needs.

The final control variable measured readiness for change. This variable is a 3-item scale measured at baseline (in prison). The three items were the following: (1) “You are tired of the problems caused by the crimes you committed”; (2) “You want to get your life straightened out”; and (3) “You will give up friends and hangouts that get you into trouble after you are released”. Responses were measured on a 4-point Likert scale ranging from strongly agree (=1) to strongly disagree (=4), with higher numbers indicating a lower readiness for change. One factor emerged with an eigenvalue of 1.63 and factor loadings ranged from 0.70 to 0.79.

### 2.3. Analytic Approach

Given the dichotomous nature of the two dependent variables—perceived and objective familial financial support—logistic regression models were used to examine the effects of LFOs on financial support from family members. State fixed effects (i.e., a dummy variable for the state where the respondent was incarcerated) were included to account for variability in state policies and procedures regarding programming, release, and court-ordered fees, as well as differences in the implementation of the SVORI across states [57]. Information pertaining to the number of respondents per state is shown in Table 1.

Additionally, we employed post-stratification weighting to address the level of attrition in the SVORI data. Post-stratification is a common method used to remedy issues of non-response in sampling and research [58], and has been previously applied to the SVORI data (see [34,57,59]). In the SVORI data, attrition is problematic since individuals who remain in the study over multiple waves may be qualitatively different from those who are more difficult to follow and attrite from the study. This loss of cases in turn may impact outcomes, such as their likelihood of receiving familial support. It is important to note, however, that prior research has found attrition in the SVORI to be random [60]. (High attrition is common in panel studies [61,62], and is not exclusive to SVORI. Other longitudinal studies examining formerly incarcerated individuals have also encountered high rates of attrition (see [63]).)

To estimate post-stratification weighting, we weighted the analytic sample to a known population [58]; in this analysis, we used the known population from the wave 1 sample where interviews took place in prison. Only 60% of the sample was retained between the first and second wave of the project, a significant attrition rate [64]. In line with prior studies employing the SVORI data [57,59], we created post-stratification weights using respondent characteristics. In the current study, prior drug use, race, and age [65,66] were identified as being related to either perceived or objective familial financial support. One hundred eighty-five strata combinations were constructed using these three variables. This yielded a final weighting variable prior to attrition.

We determined our analytic sample through the following procedures. Beginning with the 1697 individuals in the baseline sample, 713 individuals were lost to follow-up in wave 2. Our topic of study excluded individuals who recidivated by wave 2 given that the interview instruments used incomparable wording for questions about financial support for respondents who were reincarcerated versus those who remained in the community. The focus on individuals who had not recidivated may have led to selection bias where those most likely to receive familial support remained in the sample. This focus is the result of data constraints; respondents who had recidivated were not asked about their perception of available financial support from family. As a conceptual matter, being incarcerated does not preclude individuals from receiving financial support, or perceiving that such support is available, from their families (see, for example, [29,38]). Future research should work to understand familial support along the full spectrum of incarceration and reentry.

Approximately 71 respondents were excluded due to reincarceration. Our focus was on fathers, specifically fathers of children under 18 who likely require caretaking or financial support. Of the remaining 913 men in the sample, 581 respondents had children, and of those individuals, 548 had children under 18. This group of 548 fathers became our primary sample. Finally, of the 548 eligible men in the sample, 22 respondents were lost due to missing data on our key analytic variables. Accordingly, the final sample was made up of 526 formerly incarcerated fathers. (Ancillary analyses included tests of whether the results presented in this study differed for parents and non-parents. These tests showed no statistically significant differences between parents and non-parents. One reason may be because a substantial proportion of the men in the SVORI dataset are fathers.)

We tested a series of models to assess the relationship between LFOs and perceived or objective financial familial support. We began by examining a model for understanding whether there was a relationship between our first dependent variable, perceived financial familial support, and LFOs for fathers (Model 1). Next, we included two models (Models 2 and 3), which included key control variables: whether the respondent had child support obligations and the amount of time they spent with their children on a monthly basis. We analyzed these separately to determine if these conditions influenced the relationship between LFOs and perceived financial familial support. We replicated Models 1–3 with the dependent variable of objective financial familial support in Models 4–6. Finally, we assessed whether there was multicollinearity among our variables. The average VIF was 1.55, and ranged from 1.08 to 2.94, demonstrating that multicollinearity was not a concern.

## 3. Results

### 3.1. Regression Models

This study focused on whether owing court debts, or having LFOs, was related to how respondents perceived their family’s willingness to provide financial support and whether their family in fact provided financial support. We also accounted for whether parental obligations, including child support and frequent interaction with their children, explained the effects of owing court debts. Accordingly, our discussion focuses on the coefficients associated with whether the individual owed court-ordered fees and whether or not those coefficients changed after adjusting for parental obligations. Following the guidance of other scholars, we do not provide a discussion of the results associated with control variables; however, coefficients, standard errors, and significance levels for these variables are available in Table 2 [67]. In addition, though each of the regression models includes fixed effects for respondents’ state of incarceration, to save space, these coefficients are suppressed in the tables; full models are available in Appendix A Table A1.

#### 3.1.1. Dependent Variable: Perceived Available Financial Support

Previous research suggests that formerly incarcerated individuals may rely heavily on familial support. At the same time, parents’ competing needs to provide for their children and to pay LFOs may conflict with the support families provide. We started by testing whether owing court-ordered fees influenced incarcerated individuals’ perceptions of available financial support. Model 1 shows that owing court fees did not influence individuals’ perceptions of available family financial support.

We also tested whether the association between owing court fees and financial support changed when accounting for whether respondents owed child support and how frequently they saw their children. As shown in Table 2, Models 2 and 3, respectively, the inclusion of these two variables did not impact the statistical relationship between court-ordered fees and perceptions of available financial support. In short, there was no statis tical relationship between LFOs and perceived familial financial support, regardless of parental obligations.

#### 3.1.2. Dependent Variable: Receipt of Financial Support

We focused next on whether owing court-ordered fees influenced the receipt of financial support from family, as well as how, if at all, parental obligations impacted that association. The results presented in Table 2, Model 4, show that fathers who owed court fees were more likely than fathers who did not owe fees to receive financial support from their families (*b* = 1.580; *p* < 0.05). Taking the exponential of the β coefficient gives the corresponding odds ratio (OR), which is 4.853. That is, the odds that fathers who owed court fees received financial support from family were 4.853-fold greater than the odds were for fathers who did not owe court-ordered fees.

To test whether parental obligations influenced familial financial support, we accounted for whether an individual paid court-ordered child support (Table 2, Model 5). The results showed that fathers who owed court fees, when accounting for whether they paid child support, were more likely than their counterparts who did not owe court fees to receive financial support (*b* = 1.504; *p* < 0.05). The odds of receiving financial support from family was 4.497 when accounting for owed child support. Another way to test whether parental obligations explained the association between court fees and financial support was to account for whether fathers saw their children at least monthly. Here, again, as shown in Table 2, Model 6, even after controlling for participants’ contact with children, owing court fees was positively and statistically significantly associated with the receipt of financial support (*b* = 1.565; OR = 4.785; *p* < 0.05). Accordingly, even when controlling for parental obligations, fathers who owed court fees were more likely than fathers who did not owe fees to receive financial support from family.

## 4. Discussion

This study reveals that, like many criminal justice sanctions, LFOs punish not just the individual convicted of a crime but their family as well. In particular, LFOs were found to significantly predict the likelihood that fathers received financial support from family. This association between LFOs and receipt of financial support remained even after controlling for an array of key variables, including whether the father owed child support or saw his children monthly. These results suggest that fathers’ greater financial need brought on by court debts is met with greater financial support from their families. However, we found no evidence that LFOs predicted fathers’ perceptions of available financial support. This finding is perhaps unsurprising given the extent of support families tend to provide their incarcerated loved ones [29,38], which may garner expectations that this support will continue once released.

The finding that owing court fees increased the financial support of families is particularly notable given the fact that many formerly incarcerated individuals come from economically poor communities [44,68]. Accordingly, court debts may further disadvantage individuals, families, and communities. In addition, prison itself diminishes the ability to maintain and cultivate quality relationships with family members, particularly children, post-incarceration [69]. Research is needed to better understand whether LFOs and the provision of financial support after prison release impacts familial relationships. For example, it may be the case that relying on family members for financial assistance harms ties between formerly incarcerated individuals and their families. In particular, tension may arise if formerly incarcerated individuals are paying criminal justice debts and because of that are unable to contribute to other household needs. For parents specifically, having to pay court debts may mean there is less money to put towards the needs of their children (see, generally, [5]).

It is also possible that the adverse consequences of LFOs are concentrated among certain demographic groups and communities. Indeed, scholars have established that mass incarceration and its collateral consequences have been especially felt by racial and ethnic minority communities [70,71,72,73]. In the current study, focusing on the full sample may have obscured within sample differences. For example, the adverse impacts of LFOs may be greatest for Black fathers. Such a finding, when considered alongside research that has found that Black men with a criminal record are likely to be overlooked for employment opportunities [74], would suggest that it may be especially difficult for Black fathers to pay off their legal debts (see generally, [75]). Future research should investigate this possibility. Empirical attention is also needed to better understand how LFOs are assigned. Scholars have found that Black and Hispanic individuals and communities tend to be viewed as more culpable and dangerous, which can result in harsher sentencing [76,77,78]. Research is needed about whether these biases are present in decision making about who is assigned LFOs, as well as what LFO amounts are assigned.

Efforts to assuage the burden LFOs cause families of returning citizens may include reducing the imposition of court-ordered fines and fees. It also may include alternative punishments, such as community service, when individuals are unable to pay financial sanctions (see, [12,20]). However, it will also be important to focus on support efforts that can reduce the need to receive financial assistance from family. An inspection of the coefficients for the current study’s control variables (Table 2) shows that men who were employed at wave 2 were approximately 1.5-fold less likely to receive financial support than those who were unemployed. This finding suggests that perhaps employed men did not need to borrow money from family. Accordingly, promoting employment opportunities for returning citizens, may be one way to reduce the financial burden experienced by their families. Such steps, however, should extend beyond “ban the box” type policy initiatives, which in some cases may reduce job opportunities for Black applicants [75]. Rather, steps might include working to destigmatize a felony conviction or having served a prison sentence, as well as providing high-quality job training and professional development opportunities to the incarcerated population.

Though we believe that each of the implications discussed above is warranted, there are some limitations that bear mentioning and that should be considered when interpreting this study’s results. First, the current study is correlational and therefore cannot establish the causal effect of LFOs on perceived or actual support. We do, however, control for several key variables that reduce the likelihood of a spurious result. Second, prior research suggests that in some cases LFOs may increase the likelihood of familial support, while in other cases they may decrease it. Future work, including qualitative assessments, should investigate whether LFOs impact how families think about the support they give, or do not give, to their loved ones. Third, as discussed, the question about court fees did not have an exhaustive list of response options. Future studies will want to give individuals the option to report that they do not know whether they have LFOs. Fourth, this study excluded individuals who had recidivated by three months post-release. Familial support provided to these individuals may be different than it was for individuals who remained in the community. Future studies should examine familial support at all stages of the criminal justice process. Fifth, this sample is not representative. For example, less than one percent of the current sample is Hispanic/Latino/Spanish, as compared to over 18% of the general population [79]. In addition, over half of the sample participated in a reentry program, which may partially explain the higher employment rate and lower familial financial support rate than might be expected. As such, the findings may not generalize to other formerly incarcerated fathers. Each of these limitations provides avenues for researchers to build on the current study’s findings and to advance knowledge about LFOs and their impacts on formerly incarcerated individuals and their families.

## 5. Conclusions

Prior research has established that if formerly incarcerated individuals are to succeed in their reentry to society, support from their families and loved ones is needed. This need can be especially true for parents who have to navigate overcoming common barriers to reentry while also trying to meet the needs of their children. The current study finds that owing court debts when released from prison can contribute to an even greater need for financial support from one’s family. These results support arguments by scholars and advocates that LFOs can adversely impact not just the person who owes the debt but their family as well.

## Figures and Tables

**Table 1:** Descriptive Statistics for all Study Variables (*n* = 526).

Variables	Observed Mean	Standard Deviation	Weighted Mean	Standard Error
*Dependent variables*				
Perception of family financial support	0.869	0.338	83.7%	(1.1)
Receipt of family financial support	0.188	0.391	15.5%	(1.1)
*Independent variables*				
Does not owe court-ordered fees *	0.272	0.445	25.8%	(1.5)
Owes court-ordered fees	0.551	0.498	54.1%	(1.4)
Unsure/refused court-ordered fees item	0.177	0.382	20.1%	(1.3)
*Key control variables*				
Court ordered to pay child support	0.386	0.487	36.1%	(1.4)
Sees child(ren) at least monthly	0.715	0.452	67.5%	(1.4)
*Control variables*				
In a romantic relationship	0.481	0.500	43.6%	(1.6)
Age (years)	11.909	6.207	32.51	(0.007)
Black	0.597	0.491	47.1%	(0.0)
Hispanic/Latino/Spanish	0.049	0.217	0.8%	(0.0)
Other race	0.051	0.221	16.6%	(0.0)
High school diploma or GED	0.614	0.487	62.5%	(1.4)
Employed at 3 month interview	0.662	0.474	67.1%	(1.5)
Number of days incarcerated (logged)	6.341	0.998	6.204	(0.029)
SVORI experimental group	0.536	0.499	50.9%	(1.6)
Supported by employment pre-prison	0.646	0.479	69.2%	(1.5)
Supported by friends and family pre-prison	0.350	0.477	31.4%	(1.5)
Needs drug or alcohol treatment ^a^	0.190	0.393	21.7%	(1.1)
Readiness for change ^b^	−0.133	0.952	−0.153	(0.031)
Has chronic illness or disease ^b^	0.411	0.492	48.9%	(1.5)
*Fixed effects*				
Iowa *	0.095	0.294	9.6%	(0.9)
Indiana	0.118	0.323	16.8%	(1.0)
Kansas	0.021	0.143	2.3%	(0.4)
Maryland	0.129	0.336	17.2%	(1.0)
Maine	0.025	0.155	4.3%	(0.5)
Missouri	0.055	0.228	4.4%	(0.7)
Nevada	0.110	0.314	8.4%	(0.8)
Ohio	0.055	0.228	3.0%	(0.6)
Oklahoma	0.036	0.187	2.4%	(0.6)
Pennsylvania	0.093	0.291	8.8%	(0.8)
South Carolina	0.234	0.424	20.3%	(1.3)
Washington	0.029	0.167	2.5%	(0.5)

Notes: * = reference category; a = 3 months post-release; b = baseline (in-prison).

**Table 2:** Logistic Regression Models Predicting Perception of Financial Support and Receipt of Financial Support (*n* = 526).

	Perception of Financial Support			Receipt of Financial Support		
	Model 1	Model 2	Model 3	Model 4	Model 5	Model 6
	*b* (S.E.)	*b* (S.E.)	*b* (S.E.)	*b* (S.E.)	*b* (S.E.)	*b* (S.E.)
Owes court-ordered fees	−0.005 (0.352)	−0.078 (0.352)	−0.032 (0.364)	1.580 * (0.298)	1.504 * (0.297)	1.565 * (0.298)
Unsure/refused court-ordered fees item	0.307 (0.334)	0.279 (0.339)	0.446 (0.342)	0.632 (0.338)	0.559 (0.343)	0.650 (0.334)
Court ordered to pay child support		0.463 (0.241)			0.369 (0.225)	
Sees child(ren) at least monthly			0.774 * (0.211)			0.116 (0.250)
In a romantic relationship	1.666 * (0.255)	1.710 * (0.272)	1.623 * (0.258)	0.363 (0.228)	0.415 (0.228)	0.359 (0.229)
Age (years)	−0.038 * (0.016)	−0.038 * (0.016)	−0.035 * (0.016)	−0.030 (0.016)	−0.032 * (0.016)	−0.029 (0.016)
Black	−0.302 (0.244)	−0.361 (0.266)	−0.339 (0.244)	1.201 * (0.294)	1.194 * (0.292)	1.196 * (0.294)
Hispanic/Latino/Spanish	1.011 (1.019)	1.004 (1.004)	1.130 (1.062)	0.118 (0.629)	0.144 (0.632)	0.120 (0.627)
Other race	0.271 (0.336)	0.325 (0.328)	0.083 (0.337)	−0.216 (0.328)	−0.184 (0.336)	−0.217 (0.329)
High school diploma or GED	0.676 * (0.205)	0.675 * (0.205)	0.682 * (0.206)	0.212 (0.244)	0.199 (0.243)	0.215 (0.242)
Employed at 3 month interview	−0.059 (0.226)	−0.037 (0.224)	−0.012 (0.233)	−1.448 * (0.270)	−1.463 * (0.275)	−1.449 * (0.270)
Number of days incarcerated (logged)	0.481 * (0.110)	0.502 * (0.120)	0.496 * (0.110)	0.217 (0.113)	0.226 * (0.113)	0.220 (0.113)
SVORI experimental group	0.439 * (0.213)	0.426 (0.218)	0.458 * (0.215)	−0.384 (0.214)	−0.415 (0.220)	−0.379 (0.213)
Supported by employment pre-prison	−0.273 (0.256)	−0.292 (0.253)	−0.110 (0.254)	−0.128 (0.256)	−0.161 (0.256)	−0.120 (0.261)
Supported by friends and family pre-prison	−0.951 * (0.218)	−0.937 * (0.219)	−0.885 * (0.225)	0.839 * (0.217)	0.850 * (0.218)	0.846 * (0.219)
Needs drug or alcohol treatment	0.142 (0.240)	0.153 (0.240)	0.257 (0.247)	−1.019 * (0.315)	−1.031 * (0.322)	−1.010 * (0.315)
Readiness for change	−0.346 * (0.101)	−0.336 * (0.102)	−0.356 * (0.102)	−0.305 (0.163)	−0.299 (0.164)	−0.306 (0.163)
Has chronic illness or disease	0.255 (0.239)	0.243 (0.241)	0.332 (0.248)	0.168 (0.255)	0.160 (0.260)	0.173 (0.256)
*Constant*	−1.154 (0.928)	−1.449 (0.982)	−2.014 * (0.965)	−4.491 * (0.889)	−4.592 * (0.885)	−4.605 * (0.933)

Notes: Standard errors in parentheses; * *p* < 0.05; These models include fixed effects for 12 states; full models available in Appendix A Table A1.

## Data Availability

Data are available by request from the Inter-university Consortium for Political and Social Research (https://www.icpsr.umich.edu/web/NACJD/studies/27101) (accessed on 7 May 2013).

## Appendix A

**Table A1:** Logistic Regression Models Predicting Perception of Financial Support and Receipt of Financial Support (*n* = 526).

	Perception of Financial Support			Receipt of Financial Support		
	Model 1	Model 2	Model 3	Model 4	Model 5	Model 6
	*b* (S.E.)	*b* (S.E.)	*b* (S.E.)	*b* (S.E.)	*b* (S.E.)	*b* (S.E.)
Owes court-ordered fees	−0.005 (0.352)	−0.078 (0.352)	−0.032 (0.364)	1.580 * (0.298)	1.504 * (0.297)	1.565 * (0.298)
Unsure/refused court-ordered fees item	0.307 (0.334)	0.279 (0.339)	0.446 (0.342)	0.632 (0.338)	0.559 (0.343)	0.650 (0.334)
Court ordered to pay child support		0.463 (0.241)			0.369 (0.225)	
Sees child(ren) at least monthly			0.774 * (0.211)			0.116 (0.250)
In a romantic relationship	1.666 * (0.255)	1.710 * (0.272)	1.623 * (0.258)	0.363 (0.228)	0.415 (0.228)	0.359 (0.229)
Age (years)	−0.038 * (0.016)	−0.038 * (0.016)	−0.035 * (0.016)	−0.030 (0.016)	−0.032 * (0.016)	−0.029 (0.016)
Black	−0.302 (0.244)	−0.361 (0.266)	−0.339 (0.244)	1.201 * (0.294)	1.194 * (0.292)	1.196 * (0.294)
Hispanic/Latino/Spanish	1.011 (1.019)	1.004 (1.004)	1.130 (1.062)	0.118 (0.629)	0.144 (0.632)	0.120 (0.627)
Other race	0.271 (0.336)	0.325 (0.328)	0.083 (0.337)	−0.216 (0.328)	−0.184 (0.336)	−0.217 (0.329)
High school diploma or GED	0.676 * (0.205)	0.675 * (0.205)	0.682 * (0.206)	0.212 (0.244)	0.199 (0.243)	0.215 (0.242)
Employed at 3 month interview	−0.059 (0.226)	−0.037 (0.224)	−0.012 (0.233)	−1.448 * (0.270)	−1.463 * (0.275)	−1.449 * (0.270)
Number of days incarcerated (logged)	0.481 * (0.110)	0.502 * (0.120)	0.496 * (0.110)	0.217 (0.113)	0.226 * (0.113)	0.220 (0.113)
SVORI experimental group	0.439 * (0.213)	0.426 (0.218)	0.458 * (0.215)	−0.384 (0.214)	−0.415 (0.220)	−0.379 (0.213)
Supported by employment pre-prison	−0.273 (0.256)	−0.292 (0.253)	−0.110 (0.254)	−0.128 (0.256)	−0.161 (0.256)	−0.120 (0.261)
Supported by friends and family pre-prison	−0.951 * (0.218)	−0.937 * (0.219)	−0.885 * (0.225)	0.839 * (0.217)	0.850 * (0.218)	0.846 * (0.219)
Needs drug or alcohol treatment	0.142 (0.240)	0.153 (0.240)	0.257 (0.247)	−1.019 * (0.315)	−1.031 * (0.322)	−1.010 * (0.315)
Readiness for change	−0.346 * (0.101)	−0.336 * (0.102)	−0.356 * (0.102)	−0.305 (0.163)	−0.299 (0.164)	−0.306 (0.163)
Has chronic illness or disease	0.255 (0.239)	0.243 (0.241)	0.332 (0.248)	0.168 (0.255)	0.160 (0.260)	0.173 (0.256)
Indiana	0.209 (0.469)	0.277 (0.473)	0.280 (0.502)	1.594 * (0.527)	1.637 * (0.522)	1.597 * (0.527)
Kansas	−0.630 (0.732)	−0.557 (0.755)	−0.886 (0.721)	1.049 (0.825)	1.070 (0.852)	1.020 (0.822)
Maryland	0.160 (0.397)	0.301 (0.394)	0.160 (0.418)	0.659 (0.499)	0.715 (0.493)	0.642 (0.493)
Maine	−2.947 * (0.582)	−3.000 * (0.579)	−2.774 * (0.568)	1.424 * (0.673)	1.348 * (0.679)	1.456 * (0.687)
Missouri	−0.010 (0.675)	−0.018 (0.667)	−0.051 (0.708)	−1.274 (0.828)	−1.377 (0.842)	−1.286 (0.825)
Nevada	0.498 (0.662)	0.548 (0.651)	0.717 (0.705)	1.850 * (0.509)	1.880 * (0.510)	1.859 * (0.513)
Ohio	1.879 (1.126)	1.917 (1.116)	2.141 (1.201)	0.902 (0.623)	0.918 (0.615)	0.916 (0.629)
Oklahoma	−0.861 (0.714)	−0.738 (0.718)	−0.836 (0.701)	−0.396 (1.058)	−0.372 (1.059)	−0.402 (1.051)
Pennsylvania	−0.884 (0.469)	−0.899 (0.475)	−0.859 (0.501)	1.240 * (0.541)	1.215 * (0.544)	1.263 * (0.547)
South Carolina	−0.436 (0.349)	−0.298 (0.363)	−0.387 (0.377)	−0.821 (0.523)	−0.758 (0.529)	−0.806 (0.531)
Washington	−0.885 (0.522)	−1.000 (0.548)	−0.504 (0.534)	−0.566 (1.003)	−0.730 (1.004)	−0.541 (1.009)
*Constant*	−1.154 (0.928)	−1.449 (0.982)	−2.014 * (0.965)	−4.491 * (0.889)	−4.592 * (0.885)	−4.605 * (0.933)

Notes: Standard errors in parentheses; * *p* < 0.05; Iowa is the reference state.
